# Indices of comparative cognition: assessing animal models of human brain function

**DOI:** 10.1007/s00221-018-5370-8

**Published:** 2018-09-28

**Authors:** Sebastian D. McBride, A. Jennifer Morton

**Affiliations:** 1 Institute of Biological, Environmental and Rural Sciences, Aberystwyth University, Penglais, Aberystwyth, Ceredigion SY23 3FG UK; 2 Department of Physiology, Development and Neuroscience, University of Cambridge, Downing Street, Cambridge, UK

**Keywords:** Cognition, Sheep, Animal model, Brain

## Abstract

Understanding the cognitive capacities of animals is important, because (a) several animal models of human neurodegenerative disease are considered poor representatives of the human equivalent and (b) cognitive capacities may provide insight into alternative animal models. We used a three-stage process of cognitive and neuroanatomical comparison (using sheep as an example) to assess the appropriateness of a species to model human brain function. First, a cognitive task was defined via a reinforcement-learning algorithm where values/constants in the algorithm were taken as indirect measures of neurophysiological attributes. Second, cognitive data (values/constants) were generated for the example species (sheep) and compared to other species. Third, cognitive data were compared with neuroanatomical metrics for each species (endocranial volume, gyrification index, encephalisation quotient, and number of cortical neurons). Four breeds of sheep (*n* = 15/sheep) were tested using the two-choice discrimination-reversal task. The ‘reversal index’ was used as a measure of constants within the learning algorithm. Reversal index data ranked sheep as third in a table of species that included primates, dogs, and pigs. Across all species, number of cortical neurons correlated strongest against the reversal index (*r*^2^ = 0.66, *p* = 0.0075) followed by encephalization quotient (*r*^2^ = 0.42, *p* = 0.03), endocranial volume (*r*^2^ = 0.30, *p* = 0.08), and gyrification index (*r*^2^ = 0.16, *p* = 0.23). Sheep have a high predicted level of cognitive capacity and are thus a valid alternative model for neurodegenerative research. Using learning algorithms within cognitive tasks increases the resolution of methods of comparative cognition and can help to identify the most relevant species to model human brain function and dysfunction.

## Introduction

The Joint Programme of Neurodegenerative Diseases has recently recommended that alternative animal models should be developed to better recapitulate the biological complexity and clinical features of human neurodegenerative diseases (JPND Working Group 2014). Although rodent experimental models have been extremely useful in representing these diseases (Chudasama and Robbins 2006), recent scrutiny has suggested that these models do not accurately or fully represent the underlying aetiopathogenic mechanisms of the disease, thus reducing the likelihood of developing methods of prevention or attenuation (JPND Working Group 2014). In particular, rodent models lack gyrencephalic convolution as well as some of the anatomical and functional heterogeneity in sub-cortical structures (e.g., basal ganglia), seen in the human brain. Rodent models have also been criticised for their inability to model the complex neuropathological changes that occur during disease progression, especially in relation to cognitive function and aging (Perentos et al. 2015). Many of these issues are resolved using non-human primate models, but there are major ethical concerns, as well as high costs associated with using primates as models of long-term neurodegeneration (Morton and Howland 2013). In response to these challenges, new research is identifying alternative animal models of neurodegenerative diseases that may better represent the biological complexity of the human disease (Eaton and Wishart 2017). One potential method for assessing the suitability of a species to represent human neurophysiological brain function (and dysfunction) is to assess neuroanatomical attributes in conjunction with its performance within standardised cognitive tasks. Neuroanatomical attributes such as endocranial volume (EVC), gyrification index (convolution of the cortex), encephalisation quotient (a measure of brain weight relative to body weight), and number of cortical neurons (a measure of connectivity of the cortex) have previously been discussed as markers of cognitive capacity (Roth and Dicke 2005; Cairo 2011; Herculano-Houzel 2011a, b). The ability to undertake and resolve the same standardised cognitive task demonstrates a common underlying structural and computational mechanism/learning algorithm (MacLean et al. 2014). Differences in performance within the cognitive task reflect differences in the values within the learning algorithm, which in turn, reflects variation in neurophysiological complexity. Measuring both neuroanatomical attributes and cognitive performance as indirect measures of cognitive capacity, and assessing the correlation between these different measures, thus produces a potential measure of suitability of a species to represent human neurophysiological brain function (Campbell and Fiske 1959; MacLean et al. 2014).

We ask this question of comparative cognition with a particular interest in using sheep as a model for human neurodegenerative disorders. A transgenic model of Huntington’s disease has recently been developed in an attempt to recapitulate more fully (compared to rodent models) the clinical features found in the human condition (Morton and Howland 2013). There is also extensive opportunity to use sheep as models for other neurodegenerative diseases given that there are at least ten naturally occurring genetic mutations relevant to human disease (e.g., Batten disease, McArdle disease, Gaucher disease, and Alzheimer’s disease) (Perentos et al. 2015; Reid et al. 2017). On face validity, sheep have some clear advantages as a model of neurodegeneration compared to rodents, for example, a longer lifespan allowing more accurate modelling of the late onset and slow progression of HD, a gyrencephalic (convoluted) cortex, and greater functional heterogeneity of sub-cortical structures such as the basal ganglia. However, this face-value assessment may be insufficient and it may be that a more empirical approach is required to produce a greater level of certainty of model success. Here, we propose a process of cognitive and neuroanatomical comparison to other species, including humans, as a way of empirically assessing the appropriateness of this species as a model of human brain function.

## Methods

The methodological approach had four stages (1) identifying a suitable cognitive task and formalising cognitive attributes of that task in the context of a reinforcement-learning algorithm, (2) generating cognitive task data for the focal species (sheep), (3) compiling cognitive task data from studies in other species and comparing it to the sheep data, and (4) compiling species neuroanatomical brain measures and correlating with cognitive data. These stages are outlined below.

### Stage 1: choice of cognitive task and reinforcement-learning algorithm

For the purpose of identifying competent animal models of human neurological disease, performance within cognitive tasks reflective of complexity within the brain region of neuropathology is an important factor in the choice of task. Frontro-striatal systems are particularly pertinent in this respect as they are critical to a range of disorders such as Alzheimer’s and Parkinson’s diseases, obsessive compulsive disorder (OCD), as well as attention-deficit hyperactivity disorder (ADHD) (Chudasama and Robbins 2006). One of the most widely used tests in this respect is the two-choice visual discrimination-reversal task. This task recruits fundamental processes of associative rule learning, breaking, and re-establishment of associative links related to rule change and attentional set shifting, whereby prior information is disregarded to establish a new set of associative links (Bissonette et al. 2013). This task can also be described formally using a reinforcement-learning algorithm and thus can be used as a measure of specific cognitive attributes. The *Q*-learning algorithm is considered optimal for modelling reinforcement learning as it incorporates the value of actions (in terms of reward) and constants associated with rate of learning (*α*) and degree of stochasticity in making a choice (*β*) (Bai et al. 2014).

#### *Q*-learning algorithm

In the two-choice discrimination-reversal tasks, the critical measure is the number of trails taken to reach learning criterion once the reversal (of *S*+ and *S*−) has occurred, i.e., the visual stimulus that was indicative of 100% reward (*S*+) reverts to 0% reward and vice versa for the *S*−. During each trial, the animal has an expected reward value associated with an action that is linked to each visual stimulus. The speed, by which a rule change (during reversal) can be learnt, is determined by the difference in expected and observed reward values for each action/stimulus and how quickly this updates the *Q* action value. This update speed value is determined by the learning rate *α*. It is also affected by the *β* function which determines the degree of stochasticity (exploration) of different actions across different stimuli. This is particularly important at the point of reversal of the two-choice paradigm where habit formation can lead to low exploration of alternative actions to produce continued responding towards incorrect/unrewarded choices (perseveration) (Dayan and Balleine 2002). The number of trails (*N*) taken to reach learning criterion is thus determined by the inverse function of *α* and *β*, whereby the higher learning rate (*α*) will reduce the number of trials to criterion and the higher probability of choosing alternative options (*β*) (as opposed to that previously linked to the reward) will increase the chances of learning the rule change. Thus, the number of trials to criterion is an inverse function of *α* and *β*:

1 $$N=f\left( {{a^{ - 1}},{\beta ^{ - 1~}}} \right).$$

Within some discrimination-reversal paradigms, correction trials are introduced to prevent spatial bias and/or perseveration on one stimulus presentation. Here, the stimuli that elicited the incorrect response are presented repeatedly until the animal or agent performs the correct response. Correction trails are most important for animals or agents prone to spatial bias and/or preservation (low *β* value) forcing them to explore, and less important for those that are intrinsically more explorative (high *β* value). Its effect on determining the number of trails to reversal criterion can, therefore, be expressed as a function of *β*. An estimated effect of incorporating correction trials into the two-choice discrimination-reversal paradigm is presented in Eq. (2):

2 $${\text{No}}.\,{\text{of}}\,{\text{trials}}\,{\text{to}}\,{\text{reversal}}\,{\text{criterion}}=f\left( {{a^{ - 1}},\beta {{(0.75\beta +0.5)}^{ - 1~}}} \right).$$

By recording the choices at each trial, the critical constants that will discern differences in cognitive capacity (*α, β*) of Eqs. 1 and 2 can be calculated. For the purposes of comparative cognition, however, these raw data and calculations are rarely presented within two-choice discrimination-reversal studies. In an attempt to counter this issue for comparative purposes, the number of trails to reach criterion during the acquisition and reversal phases will be take as a composite function *α* and *β*. Previously the reversal index (RI) has been presented as a standardised measure of the two-choice discrimination-reversal paradigm to allow comparisons between species (Rajalakshmi and Jeeves 1965):

3 $${\text{RI}}=\frac{{{\text{Trials~}}\,({\text{or}}\,{\text{errors}})\,{\text{to}}\,{\text{criterion~}}\,{\text{on~}}\,{\text{reversal}}\,{\text{learning~}}\,({\text{Trials}}\,{\text{rev}})}}{{{\text{Trials~}}\,({\text{or~errors}})\,{\text{to~}}\,{\text{criterion}}\,{\text{on}}\,1{\text{st}}\,{\text{acquisition~}}\,({\text{Trials}}\,{\text{acq}})}}.$$

Here we extend the reversal index to take into account the total number of trials required for both the acquisition and the reversal phase (Trials_acq_ + Trials_rev_):

4 $${\text{RI}}=\frac{{({\text{Trials}}\,{\text{rev}})}}{{\left( {{\text{Trials}}\,{\text{acq}}} \right)}}~\left( {{\text{Trials}}\,{\text{acq}}\,+\,{\text{Trials}}\,{\text{rev}}} \right).$$

Incorporating Eqs. (1) and (4), it follows that:

5 $${\text{RI}}=f\left( {{a^{ - 1}},{\beta ^{ - 1}}} \right).$$

And with the presence of correction trails:

6 $${\text{RIc}}\,{\text{=}}\,f\left( {{a^{ - 1}},\beta {{(0.75\beta +0.5)}^{ - 1}}} \right)$$

7 $${\text{RI}}=1.78{\text{RIc}} - 0.7553.$$

RI values will thus be used for comparative purposes between two-choice discrimination-reversal studies (as a composite measure of *α* and *β*) and adjusted according to Eq. (7) where correction trials have been incorporated.

### Stage 2: two-choice visual discrimination task

#### Animals

There are over 60 breeds of commercial sheep with a general classification of upland and lowland breeds dependent on their typical environment (Maijala 1997). To attain a mean value of ovine cognitive ability, four breeds of sheep (2 upland and 2 lowland) were used in the cognitive task [Blue-faced Leicester (BFL) (lowland), Texel (lowland, island), Suffolk (lowland), and Beulah (upland)] (Table 1). All sheep were female and aged 9 months at the start of testing (*N* = 15 for each breed). All animals were born and kept within the same lowland husbandry system at Aberystwyth University. Prior to the study, all animals lived outdoors and had received the same amount of handling as part of the routine husbandry. During the study, all animals were kept indoors in a university stock barn with free access to water and *ad libitum* hay. All animals were given a feed supplement in the form of a standard ration of 400 g cereal-based pelleted concentrate per day (Wynstay Lamb Finishing nuts, Wynstay, UK). On testing days, these pellets were provided as the food reward within the operant task. All animals came from, and were returned to, permanent stock flocks held at Aberystwyth University where the experimental work was carried out.

**Table 1:** Description of the four breeds of sheep used in the experiment

Breed	Origin of breed	Morphology	Production traits	Environment
Blue-faced Leicester	Lowland breed from Northern England	White, roman nose with long upright ears. 70–105 kg	Meat and wool	Kept on lowland on rye-grass swards and supplemented with cereal-based concentrate during winter months. Often lambed indoors
Texel	Island breed from the Netherlands	White, wide-faced with wide placed ears. 85–100 kg	Meat	Predominantly kept on lowland but will also survive on sparse vegetation. Nutrition is often supplemented with cereal-based concentrate during winter months. Often lambed indoors
Suffolk	Lowland sheep evolved from the crossing of Norfolk Horn ewes with Southdown rams in the UK	Black-faced and wide-faced with long downward ears. 95–130 kg	Meat	Kept in a lowland environment on rye-grass swards and supplemented with cereal-based concentrate during winter months. The breed is often grazed on salt marshes in certain parts of the UK. Lambed indoors
Beulah	Upland breed originating in Wales	Black- and white-speckled face. 52–86 kg	Crossed with lowland sheep to produce lambs for meat	Kept on an upland environment (hill or mountain) with forage supplemented during adverse winter conditions. Lambing typically occurs outside

#### Mobile operant cognitive testing system

**Fig. 1:**
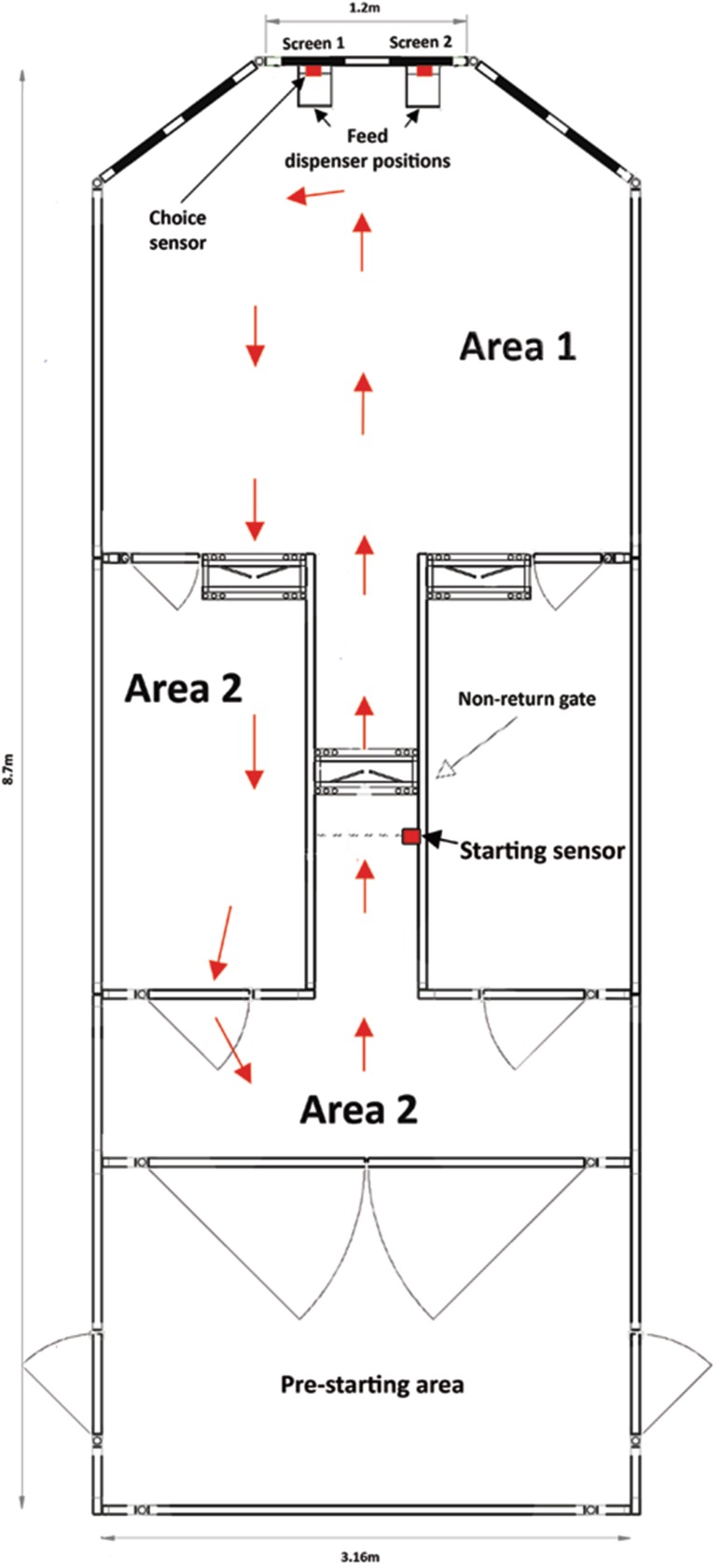
Diagram of the mobile operant system. Arrows indicate the normal route that the animal takes during each trial

We used a semi-automated operant system with capability to run various cognitive paradigms as previously described (McBride et al. 2015). In brief, the system consisted of three areas within a rectangular arena (8.7 × 3.1 m) constructed using 1 m high Paneltim plastic sheets (Paneltim, Lichtervelde, Belgium) (Fig. 1): Area 1 is the starting point where animals are held prior to beginning the test; Area 2 contains a central corridor that directs the animal towards the stimulus choice area. A one-way direction of travel through this area is maintained using one-way gates (IAE, Stoke on Trent, UK). The central corridor contained a diffuse-reflective photo-electric sensor (infrared) (Omron, Nufringen, Germany) that, when triggered, initiated the start of each trial. Area 3 is where both stimuli and reward were presented. Visual stimuli are presented via liquid crystal display (LCD) screens (Dell, UK). The animal’s choice is registered via diffuse-reflective photo-electric sensor in front of a screen. The reward (5 g of normal sheep ration in the form of pellets) was delivered to a trough directly under the screens via a feed dispenser (Quality Equipment, Woolpit, UK). Visual stimuli, sensors, and the feed dispenser are controlled using Matlab R2015a (Mathworks, UK) in conjunction with Psychtoolbox (Psyctoolbox.org) with inputs from sensors and outputs to dispensers relayed via a 12 bit USB data acquisition device (DAQ)(MCC 1208 fs) (Measurement Computing, Norton, USA).

#### Acclimation and training

In the acclimation phase, animals were habituated to the equipment. Animals were fed pellets from buckets in the operant system, first as a single group (1 × 15 min session), then as sub-groups of 7 (2 × 15 min sessions), and then groups of 3 (1 × 15 min sessions). Finally, animals were fed as pairs within the system, with pellets dispensed from the feed dispenser (1 × 15 min sessions) remotely controlled by the operator.

All animals progressed singly through four stages of training as previously described (McBride et al. 2015). The first three stages of training had the function of habituating and positively conditioning the animal to working in the operant system alone, promoting trial and error behaviour between the two points of reward delivery and introducing the animals to the one-way ambulatory circuit within each operant trial. Stage 4 training introduced the animals to the consequence of an error response. For each trial, one visual stimulus, randomly chosen from a library of 10 wingding images, was pseudorandomly presented on one screen (left or right) with simultaneous presentation of an audible tone (750 Hz, 0.5 s). Animals were required to move to the screen carrying the image to elicit a food reward. Between trials, the animal was required to exit the stimulus/reward area into the ambulatory circuit area via the non-return gate and to then return to the stimulus/reward area via the central corridor. Trials were initiated when sheep triggered the starting sensor within the central corridor. This stage had ten trials in one session. There was no time-limit on the animal moving to the correct screen. There was now, however, a consequence of choosing the incorrect screen. This led to the presentation of a high pitched audible tone (1000 Hz, 0.5 s), the image being removing, and the animal being required to reinitiate the trial by moving out of stimulus/reward area into the ambulatory circuit area and back through the central corridor. Since animals within this stage of training could now make correct or incorrect responses, the number of correct trials (animals choosing the single stimulus) was recorded. The end of the session was indicated with a prolonged low-pitched audible tone (260 Hz, 1.9 s). The total session time for each animal was approximately 6–8 min.

#### Two-choice visual discrimination task

The two-choice visual discrimination task consists of the concurrent presentation of two visual stimuli (*A, B*): one of which is assigned as the *S*+ (reward presentation) and one of which is assigned as the *S*− (no reward). Stimuli were presented concurrently on two screens (pseudorandomly; 50% left, 50% right, screens 1 and 2, Fig. 1) with simultaneous presentation of an audible tone (750 Hz, 0.5 s). For half of the subjects (pseudorandomly allocated), stimulus A was the *S*+, and for the other half stimulus *B* was the *S*+. A correct response elicited a food reward and an incorrect response resulted in the presentation of a high pitched audible tone (1000 Hz, 0.5 s) and no food reward. An incorrect response also resulted in the animal moving onto ‘correction’ trials (a repeat of the incorrect trial) until a correct response was given. Correction trials prevented strategies of side bias where the animal would consistently choose one side to attain 50% of the total reward (Horner et al. 2013). Each trial was time-limited to 45 s after which a high pitched audible tone (2250 Hz, 0.3 s) was sounded and the trial ended. Each session consisted of ten trials (stimuli presentations). The end of the session was indicated by a prolonged low-pitched audible tone (260 Hz, 1.9 s). Learning criterion was set at either 6 consecutive (*p* = 0.015) or 9 out of 10 (*p* = 0.01) correct responses. Animals continued on the acquisition learning phase until they had met criterion. Once animals had reached criterion for the first acquisition (Acq1), the *S*+ and *S*− were reversed (Rev1). Animals continued on the reversal learning phase until they met criterion. They were then tested upon a second set of novel stimuli (Acq2).

### Stage 3: compiling two-choice visual discrimination data from studies using other species

Valid comparisons of the reversal index between species are intrinsically difficult because of potential methodological differences between studies and between species. The previous work (Rajalakshmi and Jeeves 1965) has set out specific criteria by which studies could be included for comparative purposes:

1. The index must be computed from the data of the first discrimination and the first reversal phases;
2. The form of discrimination must be similar between studies (since it has been shown that values of the reversal index using the same species differ when the study uses form or shape discrimination as opposed to position or brightness discrimination);
3. The procedure and the reinforcement used for training (e.g., positive reward verses aversive stimuli) must be comparable between studies (as this has been shown to influence the rate of learning);
4. Measurement of performance must be in terms of the same unit, e.g., trials to criterion.
5. The criterion set for learning performance must be comparable. To create a common metric between these two measures, the learning criterion was set at a probability value of *p* < 0.02. Thus, for example, out of a session 10 trials, this would require a minimum of 9 correct responses *p* = 0.0098 or 6 consecutive correct responses (*p* = 0.015).
6. The reversal index for any species must be computed from a sufficiently large and representative sample of animals (*n* ≥ 10).

**Fig. 2:**
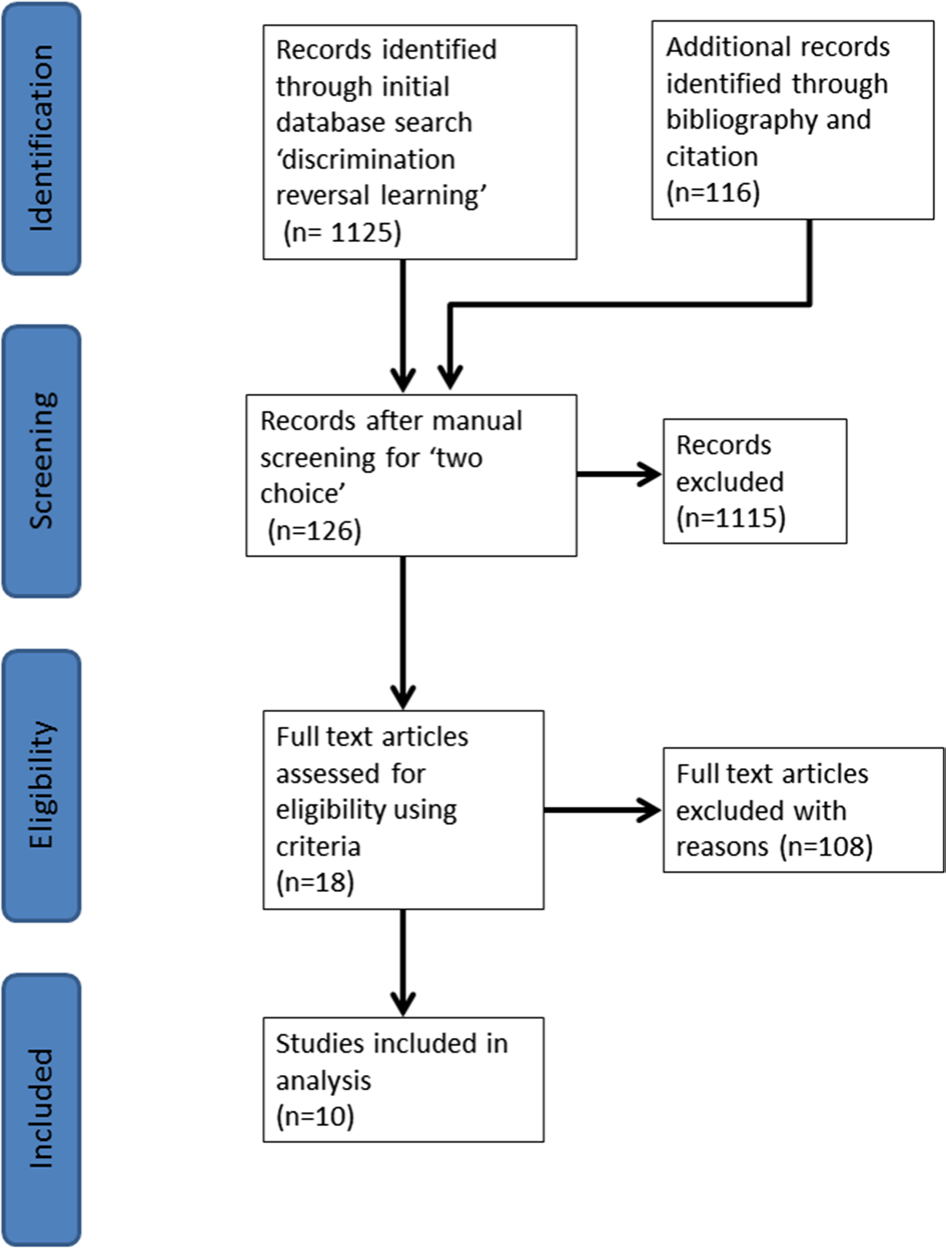
PRISMA diagram of the search process for ‘two-choice visual discrimination’ studies to be included in the correlation analyses

The Web of Science ^TM^ database was used with the search term ‘discrimination-reversal learning’. Additional studies were collated from the bibliography of studies from the primary search. Additional studies were collated from all studies that had cited the studies in the primary search. Studies were screened on the basis of (1) meeting the listed criteria (above), and (2) existence of brain morphology data for the species in question. This produced one paper per species with the exception of the mouse. In this instance, the study that had used the greatest number of animals was used as the best representation of the data point. A PRISMA diagram is presented of the search, screening, and inclusion process (Fig. 2).

The study was limited to bird and mammals and did not include fish. This was because the previous work has demonstrated that fish do not experience the same proactive interference as other species of the first acquisition memory on the subsequent reversal phase (i.e., every phase is an acquisition phase being learnt for the first time) (Gonzalez et al. 1967). The reversal index may not, therefore, be an appropriate measure of cognitive capacity for fish species.

### Stage 4: Compiling neuroanatomical measures of the brain

Brain morphology data from the literature or derived from the literature were considered to be a mean representation for each species.

Endocranial volume (EVC) data had been previously reported for a wide range of species (from rodents to primates) (MacLean et al. 2014) by dividing the brain mass by the density of fresh brain tissue (1.036). Additional values for this paper (horse, cow, pig, and sheep) were calculated using a similar approach using recorded brain mass values (Mink et al. 1981; Nieuwenhuys et al. 1998).

Gyrification index (GI) data for all species were taken from the previous studies (Brodmann 1913; Schlenska 1974; Pillay and Manger 2007; Manger et al. 2012; Zilles et al. 2013). GI is the ratio between the total outer cortical surface (superficially exposed cortex plus occluded cortex within the sulci) and the superficially exposed cortex. Since birds have no gyrification of the cortex (Jarvis et al. 2005), the GI for the two bird species (pigeon and jay) assessed in this study was set at a value of 1.

Encephalisation quotient is defined as the deviation of the regression of the brain-to-body weight ratio (Cairo 2011). It is calculated as follows:

$${\text{EQ}}=\frac{{{\text{brain}}\,{\text{weight}}}}{{\left( {0.12 \times {\text{body}}\,{\text{weigh}}{{\text{t}}^{2/3}}} \right)}}.$$

The constant indicates an important geometric relationship between volume and surface area. Jerison (1973) estimated a constant value of 0.666 for mammals and that value was used to calculate EQ values in this study. Brain mass data were sourced as previously described for ECV data.

The number of cortical neurons values for the majority of species was taken from the previous estimates (Korbo et al. 1990; Roth and Dicke 2005; Jelsing et al. 2006; Falk and Hofman 2012; Roth 2012). The number of cortical neurons for marmosets was calculated by multiplying the cortical neuron density value measured by Haug (1987) and the cortex volume value estimated by Sultan (2002). Data were not available to calculate the number of cortical neurons for the two bird species assessed in this study (pigeon and jay).

### Statistics

Each phase of the cognitive test was treated as a separate measure (Chase et al. 2012). To establish breed variation within each phase of the cognition task, data were analysed using one-way ANOVA with breed set as the between-subjects factor. Post hoc analyses between individual breeds were performed using the Bonferroni test. To assess whether neuroanatomical measures were predictive of cognitive performance, data were analysed through linear regression.

All statistical analyses were carried out using GenStat, 16th Edition. Statistical significance was set at *p* = 0.05. All data are presented as mean ± SEM.

## Ethical note

This study was approved by the University of Cambridge Research Ethic Committee and was carried out in accordance with UK laws relating to the Animals (Scientific Procedures) Act, 1986.

## Results

### Two-choice visual discrimination data for sheep

All sheep learned the visual discrimination task and the reversal. There was no significant difference between breeds in the number of trials required to reach the learning criterion during Acquisition 1 (53.5 ± 6.3 [BFL], 44.7 ± 8.4 [Texel], 35.9 ± 6.4 [Suffolk], and 34.3 ± 3.8 [Beulah]) (*F* = 1.83, *p* = 0.15). There was, however, a significant effect of breed on the number of trials to criterion during the reversal phase with the BFL sheep requiring significantly more trials than the other three breeds (65.7 ± 6.5 versus 48.6 ± 8.0 [Texel], 39.9 ± 6.0 [Suffolk], and 40.1 ± 5.3 [Beulah]) (*F* = 4.70, *p* = 0.006). Similarly, there was a significant effect of breed during Acquisition 2 with BFL sheep also requiring significantly more trials to reach criterion compared to the other three breeds (54.2 ± 8.1 versus 33.7 ± 4.3 [Texel], 28.8 ± 3.9 [Suffolk], and 32.5 ± 3.5 [Beulah]) (*F* = 5.04, *p* = 0.004). When data from all sheep tested were combined, a mean of 42.1 ± 4.4 trials were required to reach criterion for the first acquisition, 48.6 ± 6.1 trials for reversal, and 37.3 ± 5.7 trials for the second acquisition. This gave a sheep reversal index of 104.6. The breed variation in the acquisition and reversal data subsequently produced a range of reversal index values from 84.2 to 146.4 (146.4 [BFL], 101.4 [Texel], 84.2 [Suffolk], and 87.0 [Beulah]).

### Two-choice visual discrimination task data for other species

From the literature, data from two-choice visual discrimination tasks were reported for ten species and compared to the sheep data obtained from this study (Table 2). The reversal index ranged from 624.00 for mice to 15.05 in gorillas, with humans recorded at 39.05. Compared to the other ten species for which two-choice visual discrimination data were available, sheep were ranked third for the reversal index after humans and gorillas (Table 2).

**Table 2:** Comparison of reversal index values across species [data for sheep were obtained within this study (italics), all other values were derived from the literature]

Rank	Species	No. of animals	No. of trials to criterion		Correction trials	Discriminatory stimuli	Reversal index (RI)	Reference
			Acquisition (A)	Reversal (R)				
1	Gorilla	5	16	9.5	No	Random visual objects	15.05	Rumbaugh (1971)
2	Human	8	18	19	No	Random visual objects	39.05	Roberts et al. (1988)
*3*	Sheep	*14*	*42*	*49*	Yes	Random visual objects	*184.92*	This study
4	Marmoset	3	21	53	No	Random visual objects	186.76	Dias et al. (1996)
5	Dog	30	85	110	No	Size (2 blocks of different sizes)	252.35	Tapp et al. (2003)
6	Horse	17	94	114	No	Colour (black and white)	252.55	Sappington et al. (1997)
7	Jay (Western scrub and Pinyon)	10	98	134	No	Colour (red and green)	317.22	Bond et al. (2007)
8	Rhesus Monkey	12	298	194	Only for 1–2 older animals	Random visual objects	320.29	Voytko (1999)
9	Pig	16	110	180	No	Colour (black and white)	474.54	Moustgaard et al. (2004)
10	Rat	20	52	144	No	Vertical and horizontal stripes	542.76	Rajalakshmi and Jeeves (1965)
11	Mice	10	150	240	No	Random visual objects	624.00	Glynn et al. (2015)

### Correlation between external anatomical measures and reversal index

**Fig. 3:**
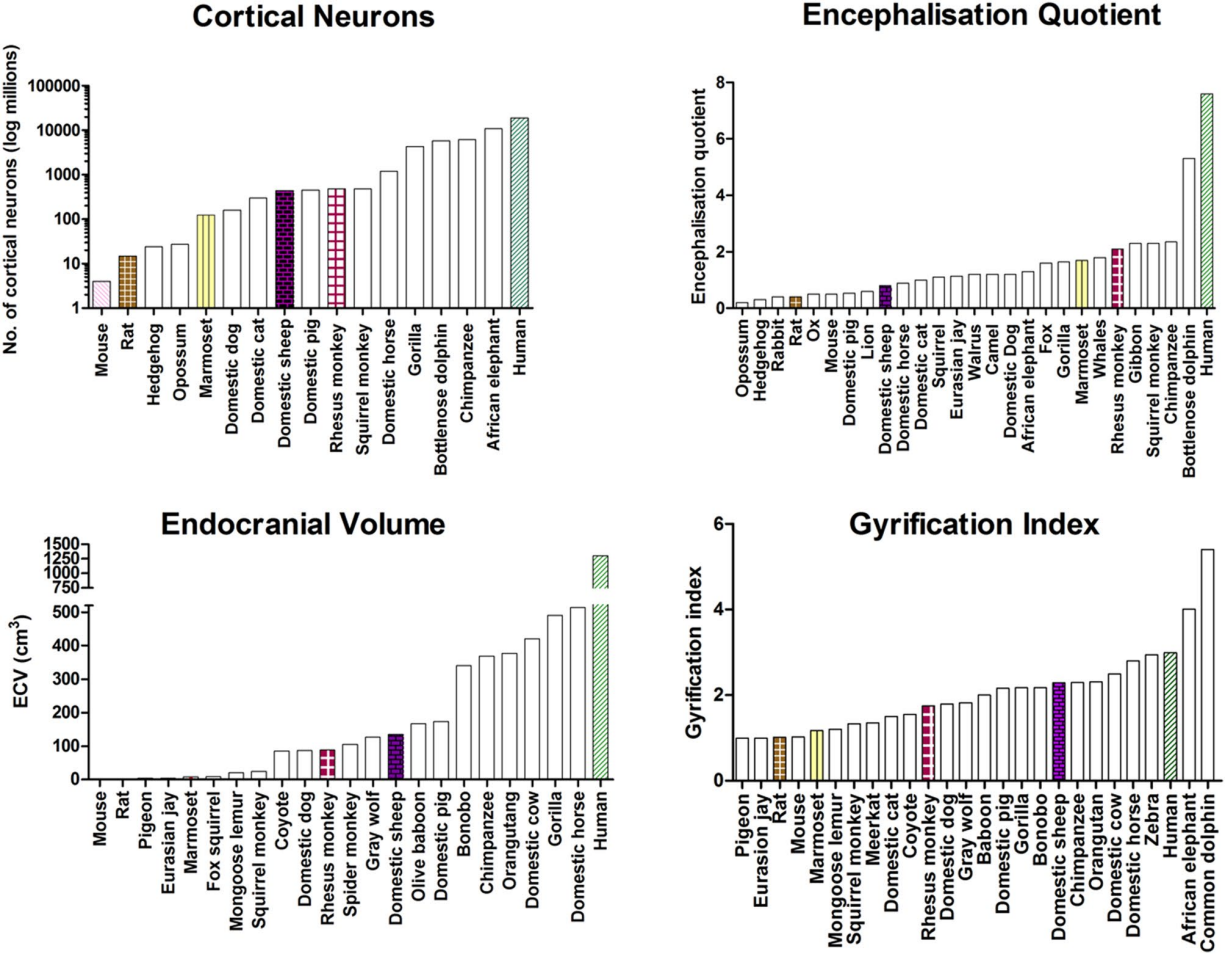
Comparison of sheep against other bird and animal species for endocranial volume (ECV), gyrification index (GI), number of cortical neurons, and the encephalization quotient (EQ)

Data for the external anatomical measures for the various species are presented in Fig. 3.

Sheep were ranked fifth for endocranial volume after humans, horses, gorillas, and the domestic pig, third for gyrification index after humans and horses, and sixth for cortical neurons after humans, gorillas, the domestic horse, rhesus monkey, and the domestic pig. Finally, sheep ranked seventh for encephalisation quotient after humans, rhesus monkeys, marmosets, domestic dogs, Eurasian Jays, and domestic horses.

**Fig. 4:**
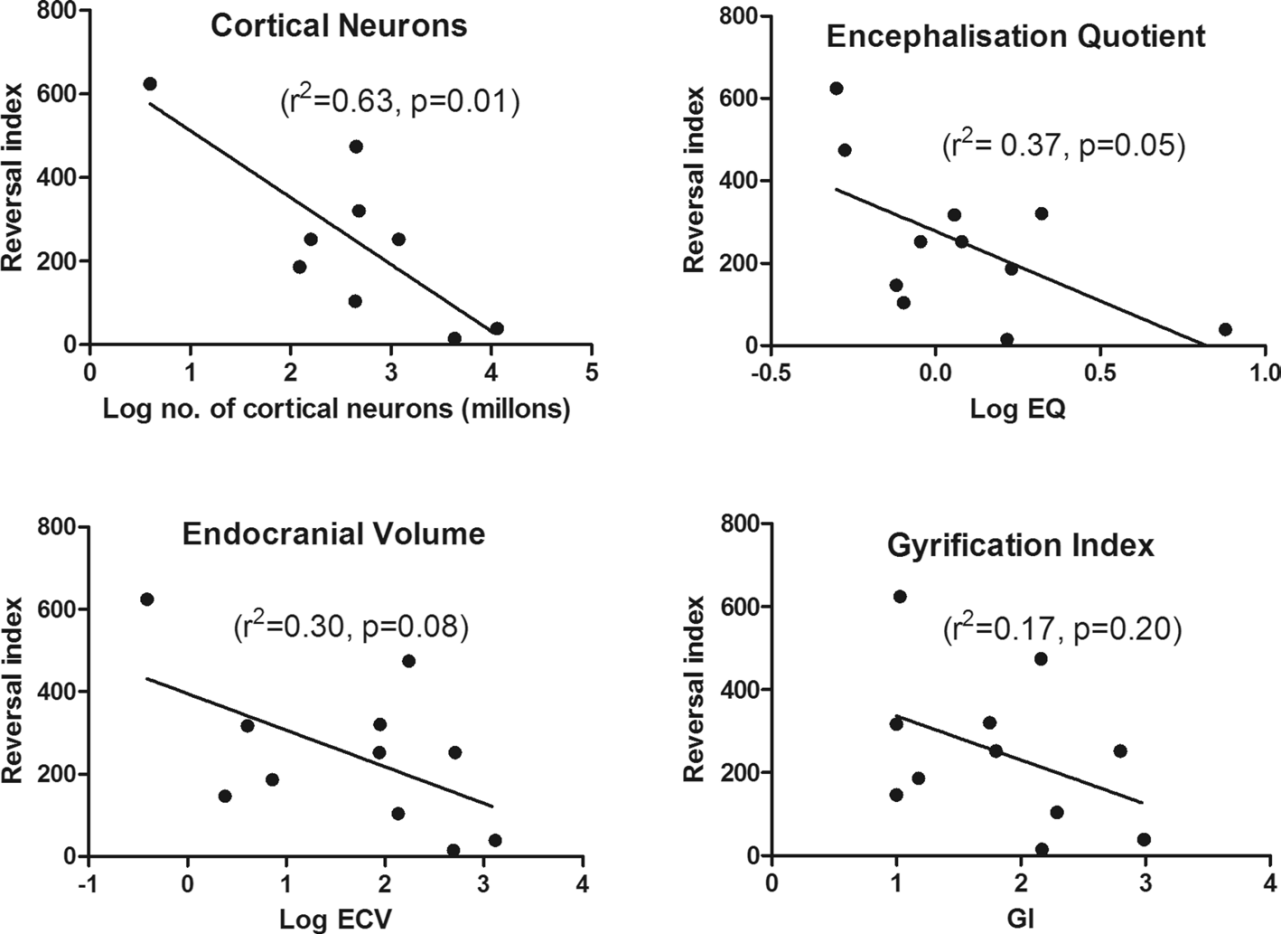
Linear correlations between reversal index and log endocranial volume, gyrification index, log number of cortical neurons, and log encephalization quotient (EQ)

Correlations between external anatomical measures and reversal index were made using the data from all species are presented in Fig. 4. The exception to this was the number of cortical neurons for the two bird species (pigeon and jay), where the data were not currently available.

The degree of correlation between the reversal index values and the data/log data obtained for the four anatomical predictors of cognitive ability are presented in Fig. 4. The number of cortical neurons was the strongest predictor of reversal index (*r*^2^ = 0.66, *p* = 0.0075) followed by the encephalization quotient data (*r*^2^ = 0.42, *p* = 0.03) and endocranial volume (*r*^2^ = 0.30, *p* = 0.08). Gyrification index (*r*^2^ = 0.16, *p* = 0.23) was observed to be a weak correlate with the reversal index values.

## Discussion

Within reinforcement-learning paradigms, *Q*-learning analysis during the task (on a trail-by-trial basis) has the potential to provide high-resolution learning data via the constants *α* and *β* within the algorithm. These constants reflect the computational complexity within specific brain regions functionally responsible for this type of learning (prefrontal cortex, amygdala, and basal ganglia) (O’Doherty et al. 2000) and, theoretically, provide a platform for comparative cognition across species. The data to allow this optimum form of learning analysis, however, tend not to be presented within reinforcement-learning studies, and thus, it is difficult to make comparisons at this level. It was postulated here that the more readily available reversal index data could act as a composite score for the *Q*-learning constants. It was also noted, however, that, as a composite measure, it may have less resolution and produce a less accurate account of the computational workings of specific brain regions. To further assess the validity of the reversal index, we correlated neuroanatomical metrics with a measure of cognitive ability. This process of convergent validity (whereby the correlation of two measures give additional support to using either measure) has previously been discussed as useful starting point for identifying a common currency of animal cognitive capacity (Campbell and Fiske 1959). The brain anatomical measures analysed within this study had a range of predictive abilities in relation to the performance within the two-choice visual discrimination task. The number of cortical neurons was found to be highly predictive of cognitive performance, EQ had moderate predictive ability, and ECV and gyrification index had no predictive ability. These data support some, but not all of the findings within a recent study by MacLean et al. (2014). In that study, MacLean and colleagues investigated the relationship between the anatomical measures of EQ and ECV and performance on two different cognitive tasks of self-control. They showed that there was a significant correlation between performance on these cognitive tasks and both EQ (*p* < 0.01) and ECV (*p* < 0.01) using data from 36 species of animals. We did not see a strong correlation between reversal index and EQ or ECV. The difference in results between their study and ours probably reflects nuances in the cognitive demands placed upon different brain regions by the different types of test. The two-choice discrimination task measures behavioural flexibility that is mediated predominantly by the prefrontal cortex in conjunction with the basal ganglia (Cools et al. 2001). The self-control tasks used by MacLean et al. (2014) are predominantly measures of compulsion and as such are likely to be mediated more heavily via sub-cortical structures within the basal ganglia (Robbins 2002). MacLean et al. (2014) speculated that the number of cortical neurons may provide a finer measure of neuroanatomical substrate and, thus, a better predictor of task performance in tasks involving executive function. This conclusion is in line with the current thinking about the number of cortical neurons as a biological correlate of cognitive capability (Herculano-Houzel 2017).

Our analysis of direct and indirect measures of cognitive ability in different species strongly suggests that sheep have the potential to be a highly useful and relevant model of human brain structure and function/dysfunction. The finding is not necessarily surprising, given the habitat and ethology of the species. Like many ungulate species in their wild state, sheep live in a complex habitat of potentially high cognitive demand. They range over extensive areas of land where they are continually required navigating to known areas of shelter, food, and water in the context of predator avoidance. Sheep are also opportunistic feeders that adapt their diet to whatever forage is available (Todd 1975). It has previously been suggested that dietary complexity is a major driver of cognitive evolution and ability for both non-human primates (Dunbar and Shultz 2007) and other non-primate species (Balda and Kamil 1989). Although lowland domestic breeds of sheep are often grazed on rye-grass clover swards with a little opportunity for diet diversity, upland domestic breeds of sheep can have access to over 22 species of forage that they will actively graze (Fraser 1998). Wild sheep of North America have been reported to forage across 267 species of plant (Wikeem and Pitt 1992). Sheep are also a highly social species and the social complexity hypothesis of cognitive capacity proposes that increased social complexity, as indexed by social group size, is also a potentially major selective pressure of cognitive evolution (Cunningham and Janson 2007). Mean wild and feral sheep group size has been reported between 3.2 and 12.7, whilst domestic sheep can be kept in groups of over 200 animals (Shackleton and Shank 1984).

## Conclusions

There is an increasing demand for better animal models of human neurodegenerative disease to represent the complex neuropathological changes that occur during disease progression, especially in relation to cognitive function and aging (JPND 2014). It is important, therefore, to be able to devise methods to assess different species in terms of their suitability as a model before committing resources to larger scale projects (Morton and Howland 2013). Detailed analysis of learning algorithms within comparable cognitive tasks across species may help to provide some of this insight. Due to cognition studies not currently providing this level of detail in the data, we used an indirect measure of learning algorithm constants, the modified reversal index. This preliminary analysis demonstrated that sheep have a high level of predicted cognitive capacity and, thus, potentially are a valid alternative to using non-human primates for neurodegenerative research. Further research using learning algorithms within cognitive tasks has the potential to increase the resolution of methods of comparative cognition as a way of identifying the most relevant species to model human brain function and dysfunction.
